# Private hospital accreditation and inducement of care under the ghanaian national insurance scheme

**DOI:** 10.1186/2191-1991-1-13

**Published:** 2011-09-01

**Authors:** Eugenia Amporfu

**Affiliations:** 1 Kwame Nkrumah University of Science and Technology, Kumasi, Ghana

## Abstract

The Ghanaian National Health Insurance Scheme pays providers according to the fee for service payment scheme, a method of payment that is likely to encourage inducement of care. The goal of this paper is to test for the presence of supplier induced demand among patients who received care in private, for profit, hospitals accredited to provide care to insured patients. An instrumental variable Poisson estimation was used to compare the demand curves for health care by insured outpatients in the public and private hospitals. The results showed that supplier induced demand existed in the private sector among patients within the ages 18 and 60 years. Impact on cost of care and patients' welfare is discussed.

## 1. Introduction

The introduction of the National Health Insurance Scheme (NHIS) in Ghana has allowed registered members to seek care at zero cost at the point of purchase and hence improved access to health care. The scheme covers about ninety five percent of common diseases in the population and patients are free to choose their own providers. The resulting increase in utilization of care caused over-crowding in public health facilities. This necessitated the accreditation of private health facilities to ease the over-crowding in the public health facilities. The government has also used demand side cost sharing measures to curb utilization rates due to moral hazard^1^. Patients of the NHIS were given attendant cards which were supposed to be filled by health facilities during each visit. The inconvenience of going to the NHIS office for new cards when those given were full was supposed to deter patients from making unnecessary visits. These measures are now being reexamined. Thus policy to reduce utilization has ignored the supply side cost sharing. Presently, a pilot study on capitation is being planned for the Ashanti region. Even though health care providers in the public and mission hospitals are salaried and hence may not have the incentive to induce demand, physicians in private hospitals are paid directly by the NHIS under a fee for service scheme and so may have the incentive to induce demand.

**Figure 1:**
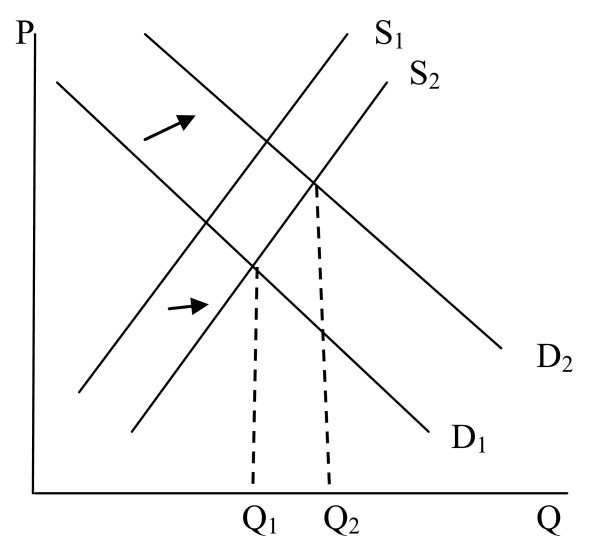
Inducement.

Supplier induced demand (SID) in the health care market refers to a situation in which the physician influences demand for his/her services in a way, according to the physician's interpretation, that is not in the best interest of the patient [1]. Given the asymmetric information that exists between the physician and the patient, with the physician being better informed than the patient, the physician has influence on the quantity of health care that the patient consumes. If this influence moves a patient towards the optimal level of consumption we have useful agency [2]. However, inducement occurs when the influence is used in a way to benefit the physician (e.g., increase in income) rather than the patient. SID involves the shifting of the demand curve [1]. Under inducement, utilization of care by the patient changes because the physician uses his/her influence to shift the demand curve to the right. This is illustrated in Figure 1. In Figure 1, an increase in the supply curve from S_1 _to S_2 _increases equilibrium quantity to Q_1 _but the resulting shifting of the demand curve from D_1 _to D_2 _further increases equilibrium quantity to Q_2_. The increase in equilibrium quantity from Q_1 _to Q_2 _is due to SID.

The definition of SID given above is consistent with the expectation that an increase in the supply of physicians and hence a reduction in the number of patients cared for by a physician gives incentive to the physician to increase demand for his/her service at a given price. This expectation is likely to occur if at a given price, the reduction in the number of patients reduces total income of the physician. Inducement then implies a positive relationship between the supply of physicians and demand for health care, a relationship referred to as the SID hypothesis.

Obviously the SID hypothesis is contrary to demand and supply analysis that shows that an increase in the number of suppliers leads to an increase in quantity demanded due to the price effect. Demand then does not shift. The shifting of demand under the influence and in the interest of the physician then challenges the basic market theory which assumes consumer sovereignty. Thus SID has important health policy implications. Since the SID hypothesis is income effect, the type of payment scheme used can affect income and hence encourage or discourage SID. Salaried physicians do not have the incentive to induce because their income is not affected by any change in the number of patients treated as a result of a change in the number of physicians and so there is no income effect. Physicians under the fee for service scheme may face a fall in income as a result of a fall in the number of patients.

SID, as described above, because it increases demand in the interest of the physician rather than the patient can be cost increasing without improving patient's health. The increase in cost can be caused by the quantity and the nature of utilization. For example, inducement in outpatient treatment of malaria can take the form of increased number of visits, diagnostic tests, and medication combination. These can both increase medical cost and impose additional cost on patients in the form of time spent in the hospital instead of other alternatives such as work. SID then causes inefficiencies and so is important to test for its existence. In a developing country like Ghana which has very limited resources for the health sector, the presence of inducement in the health sector could significantly impede development in the sector. However, to the author's knowledge, no research has been done on the possibility of inducement in the health sector. Such a research vacuum could explain why there is no mechanism in place for supply side cost sharing.

The purpose of this paper is to use data on mild malaria outpatients who lived and received treatment from health facilities in the metropolitan city of Kumasi, Ghana to test for SID in the NHIS and its effects on the cost of care and patients' welfare. To ensure consistent estimation, the instrumental variable method of estimation was used in a Poisson regression.

The rest of the paper is organised as follows. The next section describes the NHIS and explains the possibility of inducement in the scheme. Section 2.2 reviews previous studies on SID. This section is followed by section 3.1 which selects instruments for the empirical estimation. Section 3.2 gives a rationale for the use of Poisson estimation method for the study. The model is explained in Section 4.1 while Section 4.2 gives a descriptive analysis of the data. Section 5 reports the results from the regressions and section 6 concludes the study.

## 2. The NHIS, Inducement and Literature Review

### 2.1: The NHIS and the Possibility of Inducement

The NHIS was introduced in 2003 to make health care accessible to Ghanaian residents. Initially the scheme covered only services provided to registered members who received care in public health facilities. However, the resulting overcrowding in the public health facilities from increased membership led to the accreditation of some private facilities to provide care to registered members. Registered members of the scheme can now receive services that are covered by the scheme in public health facilities as well as private facilities accredited to provide care for scheme members. The accreditation of private health facilities, nationwide, started with a few facilities in June 2005 but the number has increased to 1551 facilities in 2009 [3]. This implies that the density of physicians who treat NHIS patients has increased significantly and so there could be some incentive for inducement.

The NHIS uses the fee for service payment scheme to pay health care providers. Under such payment scheme the total revenue or income to the provider of care for insured patients is positively related to the quantity of care provided. In the case of public health facilities physicians are salaried and so payment for insured patients goes directly to the hospital and does not affect the salaries of physicians. Such physicians have no incentive to increase demand when the number of physicians increases. Besides the income of such physicians does not change with the entry of new physicians hence there is no incentive for inducement. In private hospitals, however, physicians are also the owners or share holders of the facilities implying a positive correlation between physician income and the hospital revenue.

Physicians may incur psychic cost from inducement [4,1] and so are not likely to induce if it imposes high direct cost on the patient. Thus, while the psychic cost to physicians from inducement may be high when patients are poor and have to bear the direct cost of care, the psychic cost may be low when patients are insured. Inducement then is more likely to occur when patients are insured than when they are not insured. This explains why the current study used data on insured patients to test for SID.

The choice of Kumasi as the case study of this important issue is strategic. There are two cities in Ghana: Accra, the nation's capital city, and Kumasi, the commercial city and the regional capital of the Ashanti Region, the most populous region in the country. Kumasi has a population of about 2 million which is about a third of that of the Ashanti Region. The location of the city makes it a nodal city linking the northern part of the country to the south. The city has 220 health facilities including hospitals, health centers, clinics, and maternity homes. There are 44 hospitals, 36 (representing 81.8 percent) of which are private for profit. At the time of the study 26 of the private hospitals were accredited to treat NHIS patients [5].

As explained, SID is likely to occur in areas where there is a high density of physicians whose income varies with output, and in Ghana, such physicians are found in the private (for profit) hospitals. Besides, about 56.9 percent of the 160 private hospitals in Ghana are found in the two cities. With a population of about 3.5 million, Accra is bigger than Kumasi but Accra has 326 health facilities with 69 hospitals, 55 (representing 79.7 percent) of which are private for profit hospitals. Kumasi then has a larger number of private for profit hospitals relative to its population. Even if each private hospital has only one physician it means that physician patient ratio is likely to be lower in Kumasi than in Accra. This makes Kumasi a more likely environment for inducement than Accra. One cannot however, overlook the possibility of inducement in Accra, among the significant number of accredited hospitals, as well as the other urban areas in the country such as Sunyani, Takoradi, Cape Coast, etc. which are also likely to have a moderately significant density of physicians in the private sector. Ideally, then, the data should cover both cities and the municipalities as well; however, such data were not available. Even if Kumasi alone cannot be a good representation of the whole country, testing for SID in a likely area for SID to occur could provide important information to policy makers on whether or not there is the need for further research on SID in the other areas.

Malaria is a common disease in Ghana accounting for more than 40 percent of all outpatient cases in all hospitals [6]. Health facilities, public and private are often equipped, at least in terms of personnel, for the treatment of the disease. In public hospitals, independence between output and physician income provides no incentive for SID. Since these hospitals are able to treat such patients and there is no incentive for SID in the public hospitals, the demand curve for mild malaria outpatients who receive care from the public hospitals can serve as a control demand curve. The presence of SID in the private hospitals can thus be tested by comparing the control demand curve with the demand curve of similar patients who receive care from private hospitals. Given the positive relationship between output and income in the private hospitals, physicians in these health facilities may have the incentive to induce service by shifting the demand curve to the right.

Where physicians work in both types of hospitals patients could be redirected from the public hospitals to the physicians' own private hospitals by promising quicker and more efficient service. Also, referral hospitals may receive cases from other hospitals. In both instances, treatment could start in one hospital type and end in another hospital type. The detection of inducement by comparing demand curves for public and private hospitals in such a case will be difficult since the demand curves would not be for a full episode of illness for each patient. This problem was not encountered in this study because a health facility cannot attain hospital status in Ghana if it is not equipped to treat a minor case like mild malaria. Thus the study compares the demand curve for health care (hence utilization of care) of patients in public with that of those in private hospitals during an episode of mild malaria.

After controlling for patients' characteristics, if the demand curve for private facilities is located to the right of that of the public health facilities, then SID exists in the private hospitals. The location of the private hospital demand curve to the right of that of the public hospitals would represent a rightward shifting of the private hospitals demand curve, hence inducement in the private sector. A leftward shift of the demand curve could either be due to inducement by decreasing care or rationing of care [1]. Thus no conclusion can be made on inducement if the demand curve of the private facilities is located to the left of that of the public hospitals.

### 2.2: Previous Studies on SID

SID involves alteration in utilization of care due to the shifting of the demand curve. Since not all alteration in utilization involves the shifting of the demand curve, identifying SID can be challenging and earlier studies testing the SID hypothesis have encountered several problems.

In [7] the test for the SID hypothesis was done by testing for a positive relationship between demand and market output. The authors used neoclassical competitive market model to show that under the SID hypothesis, the market clearing condition makes it impossible for the demand equation to be identified. The demand equation does not have enough exogenous variables to identify structural relationships and there is high multicollinearity of the predetermined variables. In other words, the important exogenous variables such as health status, and taste for health are not observable but are correlated with the key observable exogenous variables in the demand equation. The identification problem resulting from omission of relevant variables and the use of inadequate proxy variables, severely distorts empirical tests that use cross sectional aggregate data as a result of a high correlation between the omitted variables and the market demand [1].

Researchers, aware of these limitations, tried to minimize the problem in testing for SID and had varying results. For example, [2] tested the effect of surgeon supply in 22 metropolitan areas over three years by using a two stage regression to purge the number of surgeons in the demand equation from unobservable variables omitted from the equation. His results supported SID. The approach in [8] studied the income effect of a fall in fertility rate on obstetricians and found a high correlation between a fall in within state fertility and increase in caesarean sections. The study argued that the fall in within state fertility is an exogenous shock to demand and income and so serves as a valid test for SID through income effect. The test for SID in [9] used monopolistic model and no inducement was found. The study used the population/physician ratio as a measure of exogenous income shock in a cross sectional data. A method described by [8] as dubious.

Other researchers used individual level data which is supposed to reduce the identification problem. The idea is that the unobservables at the individual level are less correlated with the market demand [1]. Another study, [10], tested for SID among contract physicians in Norway. They used physician data to compare practices of contract and salaried physicians and found no support for inducement. In order to avoid the bias caused by the identification problem, [11] randomly allocated patients and physicians in various locations while ensuring significant variation in the physician/population ratio. The study tested for changes in utilization as a result of changes in physician density. They found a significantly positive correlation between physician density and aggressiveness of proposed treatment.

The randomization in [11] may be ideal but could be too expensive. Thus the current study also used individual level data and, following [2], instrumental variable estimation to reduce possible bias that could be caused by the identification problem. The data had no variable for physician density. Thus physician density was incorporated into the study by the use of data in a period and a city with high physician density. The study tested the SID hypothesis by comparing the demand curves of patients treated by physicians in the public facilities with those treated by physicians in the private facilities in a period when the density of NHIS physicians in private hospitals was very high. The demand equation estimated in the study had number of visits to the hospital during an episode of mild malaria as the dependent variable and a dummy variable for hospital type where care was received, in addition to patients characteristics as the independent variables. Since the number of visits is count data, the Poisson estimation method was used.

The hospital type dummy variable equaled one if the hospital in which the patient received care is private and zero otherwise. Since initial visit is under the patient's influence the hospital dummy represents the choice hospital by the patient. Hospital choice is observable and is correlated with severity of illness (which is unobservable) such that sicker patients are likely to choose high quality hospital than less sick patients [12]. Private hospitals, because they compete with each other, are likely to have shorter waiting period, and give better attention to patients than public hospitals. In addition, severely ill patients are likely to make more visits to the hospital than the less severely ill patients. Mild malaria could have a continuum of degrees of severity. Thus severity of illness is supposed to be an exogenous variable in the Poisson equation but is omitted from the equation because of the difficulty of finding an appropriate proxy. This implies a correlation between the hospital type dummy and the error term in the Poisson equation leading to biased estimation. Instrumental variable estimation is thus required to purge the hospital dummy from severity of illness.

## 3. Methodology

### 3.1: Selection of Instrument

A popular instrument, as shown in [12], used for hospital choice in such estimation, is distance between the patient's home and the hospital. For distance to be a valid instrument it should be highly correlated with the hospital choice and uncorrelated with severity of illness. Distance is an important factor in hospital choice in that people are likely to choose the hospitals that are located close to their homes. In addition, one can be severely ill regardless of where the person lives in relation to the location of the hospital. Hence distance to the hospital is not correlated to severity of illness. Distance then is a determining factor in hospital choice was used as an instrument. This variable is obtained by computing, for each patient, the distance between each hospital and the patient's address, regardless of the hospital where care was received.^2 ^Patient's address here represents patient's area of residence.

For the present study the variable of interest is distance as a determining factor in choosing to visit a private hospital. Thus, the number of instrumental variables equaled the number of private hospitals in the data. An important characteristic of the metropolitan city under study is heavy traffic congestion. Hence an important determining factor for travelers within the city is travel time rather than distance. Depending on the location of the hospital in relation to the patient's home, a nearby private hospital could have a longer travel time, during rush hours, than one that is further away. Outpatient visits to the hospitals in the metropolitan city are usually made during regular working hours and patients have to travel early to the hospitals, during rush hours, otherwise they may wait for too long in the hospital. Travel time, rather than distance, was thus used as an instrument. The data had two private hospitals and hence two instruments were used. To ensure the instruments were able to purge the hospital dummy the partial R square test proposed by [13] was used to test for weak instrument.

### 3.2: The Rationale for Using Poisson Regression

The Poisson method of estimation was used instead of the linear estimation used by previous studies (e.g., [9,10,14]). The validity of Poisson method of estimation comes from the nature of number of visit as count data. Utilization, measured as the number of visits to the hospital, is influenced by both the patient and the physician. The initial visit is mostly influenced by the individual's subjective evaluation of his/her health need and the accessibility of professional care [15]. Follow-up visits are mostly influenced by the physician. Thus the variables that affect the initial visit may be significantly different from those that affect follow-up visits. As noted in [15], linear estimation does not take into account the two forces that drive the number of visits and hence can produce unreliable results. In addition count data have no negative values; e.g., a patient cannot make negative number of visits. The functional form of linear model does not restrict predicted values to be positive and so it is possible to get negative predicted values. Some studies (e.g., [10] tried to solve this problem by using the natural log of the dependent variable; however such a method makes the interpretation of the results less intuitive.

For the Poisson regression to be valid for the estimation, two assumptions have to hold. First, the probability of a visit occurring during the observation period should be constant and, second, the probability of a visit in any time period is independent of the probability of a visit in another time period. The type of data used for the study was individual data on low risk malaria outpatients and the count data were on the number of visits to the hospital in the first half of 2009 during an episode of illness. The probability of visiting the hospital is determined by patient's subjective evaluation of health needs as well as the physician's factors such as style of practice, and income factors. Such a probability function is not likely to change if the factors that determine it are constant. The duration of mild malaria is not likely to exceed two weeks. Patient's subjective evaluation of health needs, which influences the decision for initial visit, is not likely to change easily over time. In addition, there was no change in policy that could affect physicians' style of practice and income during the study period. Hence, factors that influence the physician's decision for follow-up visits are likely to remain constant within the episode of illness. The probability of visits, then, was not likely to change over the study period implying that the first assumption holds. Nevertheless it is important to perform an over dispersion test to ensure both assumptions hold.

The decision to make an initial visit is determined by the patient's evaluation of illness and the need for care but not on previous visits to the hospital. Neither does the physician's decision for follow-up visit depend on previous visits. If the physician's decision is based on previous visits then visits cannot be independent and so cannot follow the Poisson distribution. It is therefore important to test for over dispersion (or under dispersion) to ensure visits are not correlated. The test for over dispersion is also a specification test to ensure consistency and efficiency of estimated coefficients [16]. Thus, a test for over dispersion as specified in [16] was performed.

The test statistic for the over dispersion test was 1.782 with a p-value of 0.75. The null hypothesis of no over dispersion was thus not rejected. This confirmed the intuition that the data were suitable for the Poisson regression and hence the two assumptions for Poisson distribution hold. The Poisson regression has been used in earlier studies to estimate demand regressions using various count variables. Example, [17] used the number of hospital stays, [18] used number of specialist visits, and [19] used number of visits to the doctor.

To confirm the need for the instrumental estimation the over dispersion test was repeated without use of instrument and the test statistic was -8.693 with a p-value of 0.00 hence rejecting the null of no over dispersion. Thus the two stage method of estimation was used, with the first stage being a logit regression to purge the hospital dummy and the second stage being a Poisson regression with predicted values of the hospital dummy.

## 4. Estimation

### 4.1: The Model

The estimation is based on comparing the demand curve of patients in the public hospital with that of those in the private hospital. As discussed, the physicians in the public hospitals have no incentive to induce demand as a result of the independence between their income and output. After controlling for the characteristics of patients, the difference between the quantities consumed in the two hospital types would be a good estimation of the inducement in the private hospital.

As already mentioned, a two stage estimation procedure was used. The first stage was to obtain predicted values for choice hospital by logit estimation of the treatment equation and the second stage was estimation of the Poisson equation. The treatment equation was:$X_{4i}=\alpha_{1}+\alpha_{2}Z_{i}+\alpha_{3}Q_{i}+u_{i}$ where *X_4i _*is the dummy variable for hospital type and it equals one if the hospital where the patient received care is private and zero if it is public. The *Z_i _*is a vector of travel time variables. Since there are two private hospitals there are two travel time variables. The probability mass function for Poisson distribution for the number of visits to the hospital during an episode of mild malaria is:

(1) $$\mathrm{Pr}\left(y\right)=\frac{e^{-\mu }\mu^{y}}{y!}$$

where *μ *is the intensity parameter or the expected number of visits by a patient and *y *is the number of visits by patient within an episode of illness. The Poisson regression is obtained from the distribution by parameterising exponentially the relationship between *μ *and the exogenous variables: $\mu_{i}=\exp\left(x_{i}^{\prime }\beta \right)$. For the purpose of this study $x_{i}^{\prime }\beta =\beta_{1}+\beta_{2}X_{2i}+\beta_{22}X_{2i}^{2}+\beta_{3}X_{3i}+\beta_{4}{\hat{X}}_{4i}+\beta_{5i}X_{5}+v_{i}$ where *X_2i _*represents age in years of individual *i, X_3i _*is a gender dummy variable which equals one for a female and zero for a male; ${\hat{X}}_{4i}$ is the predicted values of the hospital dummy variable. Finally, *X_5i _*is a dummy variable which equals one if the patient lived in an affluent area and zero otherwise. There was no information on patients' education and income which are important determinants of health care consumption and hence this dummy variable served as a proxy for education and income. In general, people who live in affluent areas of the city are likely to be educated and have high income than those who live in ghettos. This kind of proxy has been used in previous studies (see, e.g., [20]).

A unique characteristic of the Poisson distribution is that its mean (*μ*) equals its variance. Thus the mean of the number of visit equals *μ *which is also the variance. This implies that with the exponential parameterization the variance is exp(*x'β*). The Poisson regression is thus heteroskedastic and so the standardized error estimation was used to correct for heteroskdasticity^3^.

### 4.2: Data Description

The data used for the study were on NHIS outpatients with mild malaria from four hospitals: two public hospitals and two private hospitals, in the Kumasi metropolitan area in Ghana in the first half of 2009. The sample size, after removing all the observations with missing information, was 2,045. Information on patients included age, gender and address. Information on address was used to compute the travel time variables for the instrumental variable estimation.

Even though the sample size for patients was large, the number of hospitals forms only a small percentage of the number of public and private hospitals in the Kumasi metropolitan area. The reason for such a small number of hospitals comes from the difficulty of obtaining data from these hospitals. Private hospitals were reluctant to disclose information and even where allowed, data on patient information had to be recorded manually from the hospital records, a procedure that is time consuming.

Nevertheless, the results of the study could be a good representation of the metropolitan area and the even the nation as a whole. The reason is that the regression controlled for patient characteristics that can affect the utilization of care. After such a control, the only difference in utilization that existed between the patients of the two hospital types was the differences in the style of practice. Given that physicians that treated the mild malaria in both hospitals types were general practitioners implying no difference in specialization, any systematic difference in style of practice between the two hospital types is likely to be driven by the difference in the payment schemes that has been explained. Thus the data represent well a typical difference in health care utilization between private and public hospitals in the country.

A weakness of the data used is lack of information on whether or not patients are self employed. According to [21], income is an important determinant of demand for health care because rich people have a high opportunity cost of waiting [21]. However, studies have shown that self employed individuals, regardless of income, i.e., whether they are petty traders or big business personnel, have high waiting cost than those who are salaried. Self employed individuals, especially petty traders, who cannot contract their trade to others during the time spent in the hospital may have to lose a whole day's income depending on the amount of time they spend in the hospital. Such individuals are less likely to visit the hospital than those that are employed by others and do not lose income for taking time off to go to the hospital. A variable for the self employment status of the patient then should be included in the equation.

Because private hospitals are likely to have shorter waiting period than public hospitals, all things being equal, the self employed are more likely to choose private hospital than public hospital. This implies that the variable for self employment status which is omitted from the estimation equation is correlated with hospital choice through their correlation with waiting period which is not observable. All things being equal such a condition would lead to biased estimation of the regression equation. However, such a bias is not likely to occur in the estimation in this study because of the method of estimation used. The instrument used for hospital choice, travel time, is highly correlated with hospital choice but uncorrelated with severity of illness as well as waiting period. Hence, while the dummy variable for private hospital where care was given is correlated with waiting period and hence with patient's self employment status, the predicted values of the dummy used for the estimation is not correlated with patient's profession. This removes any possible bias.

An important advantage of the data is that all the patients lived in the same city as the hospitals and there is a high density of hospitals. Thus while travel time affects the choice of hospital, it does not affect the number of visits after a hospital has been chosen. Travel time therefore is not an exogenous variable in the Poisson regression hence validating travel time as an instrument for hospital choice. Table 1 gives a summary of the data.

**Table 1:** Data Summary

	Public	Private	Total
Sample	1587	458	2045
Age	2.5	2.1	2.4
Gender			
• Male	60%	45.2%	40.9%
• Female	40%	54.8%	59.1%
Area of residence			
• Affluent	82%	78.6%	81.7%
• Ghetto	18%	21.4%	18.3%
Number of Visits	2.06	1.32	1.89
Hospital characteristics			
• Daily Outpatients	285	280	282.5
• Outpatient Doctors	3	2	2.5
• Outpatient nurses	9	8	8.5

As shown in Table 1 about 22 percent of the patients received treatment from private hospitals and the patients are on average under five years of age. The ages ranged from 3 months and 102 years with a standard deviation of about 18.9 representing a wide dispersion. The data also show that more males go to public hospitals than private hospitals. However, with an average age below five years the hospital choice is likely to be made by parents, mostly mothers. Majority of the patients lived in affluent areas of Kumasi and so are likely to be educated or on the part of children, their parents are likely to be educated. On average the number of visits per patient in the public hospital exceeds that of those in the private hospital. On average about 283 outpatients are treated in each of the hospitals that are used for the study. These patients are treated by about three doctors and nine nurses. Since these are raw data one cannot conclude inducement without regression estimation.

## 5: Results

The result on the test for weak instrument had a partial *R *squared of 0.3 which is significantly high and thus confirms that the instruments used are not weak. The results from the logit estimation for the treatment equation are reported in Table 2. As expected, the sign of both travel time coefficients was negative implying that patients were likely to choose private hospitals as the travel time to the hospitals fell. The coefficients are statistically significant at 5 percent significant level, confirming the results of the test for weak instruments.

**Table 2:** Results from the Logit Regression

	Estimated Coefficients
Gender	-0.048 (0.824)
Age	0.007 (0.268)
Residential	1.898 (0.000)
Travel time to private hospital 1	-0.230 (0.043)
Travel time to private hospital 2	- 0.080 (0.000)
Constant	-4.644 (0.000)

Dependent variable = private hospital dummy. *P-values are in brackets*

Results from the Poison regression are reported in the second column of Table 3:

**Table 3:** Results from the Poisson Regressions

	Estimated Coefficients	Estimated Coefficients (with interaction)
Gender	0.684 (0.000)	0.028
Age	0.019 (0.000)	
Age^2^	-0.00001 (0.000)	
Residential	1.59 (0.000)	0.003
Private Hospital	-0.981 (0.000)	-0.592
Active age group		-0.036
Interaction of active age and private hospital		0.127
Constant	-1.45 (0.00)	0.589

Dependent variable = number of visits to the hospital

The results from the Poisson regression show that females were more likely to make visits to the hospital than males. The number of visits also increased with age but at a decreasing rate. Those who lived in affluent areas in the city and thus those that were likely to be educated and had high income were likely to make more visits to the hospital than those who lived in ghettos. This is consistent with [21] results that high income earners are likely to increase their demand for health care, in the presence of full health insurance, if there is low substitution between healthcare and the other consumption goods. Malaria is best treated by health care and so high income earners are better off visiting hospitals for treatment than other alternative treatment. The coefficient of private hospital is negative,-0.981, implying that patients make fewer visits to the private hospital than the public hospital. Hence there cannot be any conclusion of inducement in the private hospital.

However, a close look at the data in Table 1 shows that the patients were on average less than five years old and so required their parents' help to commute to the hospital. Inducement of such patients then can have a high psychic cost to the physician. As explained in [4] and [1], physicians engage in inducement to maximize utility function which increases in income but decreases in psychic cost. Thus if there was any inducement in the private sector it is likely to occur among the more active age group. The voting age in Ghana is eighteen and retirement age is sixty. Patients within this age range are likely to be able to make hospital visits without much inconvenience. The Poisson regression was thus rerun after including a dummy variable which equaled one if the patient's age was between 18 and 60 inclusive and zero otherwise. This dummy was interacted with the predicted values of the private hospital dummy. The sign of the coefficient of this interaction variable would be an indication of inducement.

The results are reported in the third column of Table 3. The coefficient of the interaction dummy variable was positive and statistically significant. Using the standard interpretation for a model with conditional exponential mean, the number of visits for patients in the active age who received care in a private hospital exceeded that of those in the inactive age who received care in the public hospital by 0.127*exp(*x'β*) = 0.127*1.6966 = 0.22 visits. The results also imply that the number of visits for the active age group in the private hospitals exceeded that of the inactive age in the public hospitals by 12.7 percent.

The coefficient of the private hospital dummy for private hospital was still negative, -0.592, meaning that after controlling for the active age group in the private hospital, the demand curve of patients in the private hospital was located to the left of that of those who received care in the public hospital. Again after controlling for patients' characteristics and active age group in the private hospitals, the number of visits of the active age patients, trailed that of the inactive age patients in the public hospital by 3.6 percent. It follows that the active age group in the private hospitals made more visits to the hospitals then the inactive age group in the private hospitals and the active age group in the public hospital. Thus, after controlling for patient's characteristics, the number of visits of the active age group increased as one moved from public to private hospital. The opposite was however, the case for the inactive age group. Again the leftward shift of the demand curve of the inactive age group in the private hospital could be due inducement in the form of reduced utilization or due to rationing of care so no inducement conclusion could be drawn. Thus without taking psychic cost of physicians into account it would have been impossible to identify inducement of the active age group who received care in the private hospitals.

The result in this study is important because unlike previous studies such as [10], this study did not stop to conclude that there was no SID after testing for SID with the general sample. Categorizing the patients guided by psychic cost theory has revealed the existence of SID among the active age group.

## 6: Conclusion

This study has shown that inducement is practiced in the private hospitals, with NHIS accreditation, on NHIS patients in the active age. Patients in this age group who visited private hospitals were likely to be asked by physicians to make additional visits, which were unnecessary, to the hospital. Given that these patients may have to leave their work or school in order to visit the hospital, inducement among this age group could impose a high indirect cost on the patients.

Patients in the inactive age group (less than 18 years old and more than 60 years old) in the private hospitals, made fewer visits than those in the public hospitals. Thus inactive age group patients consumed less care in the private hospitals than the public hospitals. The lower consumption of care by this group of patients could be either due to inducement in the form of reduced utilization or due to rationing of care. If the inducement among the patients in the active age caused cost to increase, the reduced utilization among the inactive age group in the private hospitals could be cost reducing, so it is not clear the extent of inducement on the cost of care borne by the NHIS. However, whether the reduced care among those in the inactive age is due to inducement in the form of reduced utilization or rationing, these patients are made worse off compared to those within the same age group in the public hospitals. Thus NHIS patients in the private hospitals are made worse off than those in the public hospitals. While those in the active age receive too much care, those in the inactive age receive too little care.

To reduce inducement, the payment scheme could be changed from fee for service to prospective payment or a combination of both, a strategy that has been shown to be more effective in inducing the desired behavior rather than when used separately. Prospective payment schemes, be it capitation or budget allocation could have the disadvantage of underutilization and so are only likely to provide efficient utilization when combined with fee for service or its equivalent as well as monitoring.

The data used for this study came from one metropolitan area in Ghana where physician density is likely to be highest in the country and so inducement is very likely to occur. Thus before any general policy to reduce or prevent inducement is implemented an extensive research that covers the other metropolitan city in the country plus other municipalities would be required. The current research has raised the awareness of the existence of inducement and the need to address it through further research and implementation of policies to reduce it, if found to be an extensive problem.

## Competing interests

The authors declare that they have no competing interests.

## End Notes

^1^Moral hazard refers to the tendency of insured patients to purchase health care because price is paid by someone else, i.e., it is the substitution effect of spending on health care due to low price. This type of moral hazard can be referred to as ex post moral hazard.

^2^See [12] on more on the validity of distance as an instrument.

^3^See [16] for the standardized method of estimation.

## References

[B1] McGuire TG Culyer AJ, Newhouse AP Physician Agency Handbook of Health Economics 2000 1A 461 536

[B2] Fuchs VR The Supply of Surgeons and the Demand for Operations The Journal of Human Resources 1978 35 56 722069

[B3] Peace FM Online NHIS managers urged to improve enrolment 2009 http://news.peacefmonline.com/health/200911/32667.php

[B4] Evans R Perlmaned M Supplier-induced Demand: Some Empirical Evidence and Implications The Economics of Health and Medical Care 1974 London: Macmillan 162 173

[B5] NHIS NHIS Accreditation for Healthcare Providers (1st Batch Results) http://www.nhis.gov.gh/?CategoryID=158&ArticleID=1106

[B6] Daily Graphic 17000 Die a Year from Malaria in Ghana Daily Graphic, posted on Wings of Freedom, November 05 2005 http://wingsoffreedomandjustice.blogspot.com/2005/11/17000-die-year-from-malaria-in-ghana.html

[B7] Auster RD Oaxaca RL Identification of Supplier Inducted Demand in the Health Care Sector Journal of Human Resources 1981 16 327 342 10.2307/145624 7264294

[B8] Gruber J Owings M Physician Financial Incentives and Caesarean Section Delivery RAND Journal of Economics 1996 27 99 123 10.2307/2555794 10160536

[B9] Green J Physician-Induced Demand for Medical Care The Journal of Human Resources 1978 13 21 33 722067

[B10] Grytten J Sørensen R Type of Contract and Supplier-Induced Demand for Primary Physicians in Norway Journal of Health Economics 2001 20 3 379 93 10.1016/S0167-6296(00)00087-4 11373837

[B11] Hemenway D Fallon D Testing for Physician-Induced Demand with Hypothetical Cases Med Care 1985 23 4 344 349 10.1097/00005650-198504000-00006 3990389

[B12] Gowrinsankaran G Town RJ Estimating the Quality of Care in Hospitals using Instrumental Variables Journal of Health Economics 1999 18 747 767 10.1016/S0167-6296(99)00022-3 10847933

[B13] Bound J Jaeger DA Baker RM Problems with Instrumental Variables Estimation when the Correlation between the Instruments and the Endogenous Explanatory Variables is Weak Journal of the American Statistical Association 1995 90 443 450 10.2307/2291055

[B14] Peacock SJ Richardson J Supplier Induced Demand: Re-examining Identification and Mis-specification in Cross-Sectional Analysis European Journal of Health Economics 2007 8 267 277 10.1007/s10198-007-0044-7 17401594

[B15] Chi C An Event Count Model of Studying Health Services Utilization Medical Care 1998 36 12 1639 1659 10.1097/00005650-199812000-00003 9860054

[B16] Cameron AC Travedi PK Microeconometrics Methods and Applications 2005 Cambridge: Cambridge University Press

[B17] Deb P Trivedi PK Demand for Medical Care by the Elderly: A Finite Mixture Approach Journal of Applied Econometrics 1997 12 313 336 10.1002/(SICI)1099-1255(199705)12:3<313::AID-JAE440>3.0.CO;2-G

[B18] Pohlmeier W Ulrich V An Econometric Model of the Two-Part Decision Process in the Demand for Health Journal of Human Resources 1995 30 2 339 361 10.2307/146123

[B19] Cameron AC Travedi PK Milne F Piggott J A Microeconomic Model of the Demand for Health Care and Health Insurance in Australia Review of Economic Studies 1988 55 85 106 10.2307/2297531

[B20] Amporfu E Quality Effect of Early Discharge of Maternity Patients: Does Hospital Specialization Matter? Forum for Health Economics & Policy 2008 11 2 http://www.bepress.com/fhep/11/2/11

[B21] Grossman M Newhouse P, Culyer AJ The Human Capital Model of the Demand for Health Handbook in Health Economics 2000 North Holland: Elsevier 347 408

